# No difference in anxiety symptoms between women with unilateral and bilateral ovarian endometriosis: a prospective cross-sectional study

**DOI:** 10.1080/07853890.2026.2711545

**Published:** 2026-08-04

**Authors:** Huiyan Feng, Wenwei Li, Qingjian Ye

**Affiliations:** Gynecology Department, The Third Affiliated Hospital of Sun Yat-sen University, Guangzhou, Guangdong Province, China

**Keywords:** Ovarian Endometriosis, unilateral, bilateral, Anxiety, Depression, Sexual dysfunction, Chronic pelvic pain

## Abstract

**Objective:**

This study aimed to investigate whether women with unilateral and bilateral OMA differ in anxiety symptoms, and secondarily in depressive symptoms and sexual dysfunction symptoms, and to evaluate the independent contribution of chronic pelvic pain to psychological outcomes.

**Study Design:**

This study, a prospective, cross-sectional study, included 149 women aged 20–35 who had OMA but were not pregnant and had a desire to become pregnant. There were 79 women with unilateral OMA and 70 women with bilateral. The Zung Self-Rating Anxiety Scale, Self-Rating Depression Scale, and Female Sexual Function Index were administered at enrolment. Multivariable linear regression models were adjusted for age, BMI, chronic pelvic pain, dysmenorrhoea, primary infertility, and medication use. Post-hoc power analysis was performed.

**Result:**

The average age of the patients was 27.45 ± 3.44, with an average BMI of 19.46 ± 2.28. 29.53%(44/149) of the patients had mild or moderate anxiety, while 38.92%(57/149) had mild or moderate depression. Among the 103 patients with a history of sexual activity, 67.96%(70/103) had sexual dysfunction. In multivariable analyses, cyst laterality was not significantly associated with anxiety symptoms (adjusted B = −2.58[−5.19, 0.03], *p* = 0.052), depressive symptoms (adjusted B = −1.86[−5.25, 1.53], *p* = 0.279), or sexual dysfunction symptoms (adjusted B = −0.91[−2.01, 0.19], *p* = 0.072). Chronic pelvic pain independently predicted anxiety symptoms (*p* = 0.01) and depressive symptoms (*p* = 0.025). Post-hoc power analysis indicated an achieved power of 50% for the primary SAS comparison.

**Conclusion:**

In this study, the prevalence of anxiety, depression and sexual dysfunction was high in OMA, but there was no significant difference between unilateral and bilateral OMA. Chronic pelvic pain, rather than cyst laterality, independently predicted psychological distress. These findings underscore the importance of comprehensive pain assessment and routine psychological screening for all women with OMA.

## Condensation

### Tweetable statement

Women with ovarian endometrioma experience high rates of anxiety, depressive, and sexual dysfunction symptoms, regardless of cyst laterality. Chronic pelvic pain, not cyst location, independently predicts psychological distress in this population.

### At a glance

A

This study aimed to examine whether the laterality of ovarian endometrioma (unilateral vs. bilateral) is associated with differential levels of anxiety, depressive, and sexual dysfunction symptoms, and to evaluate the independent contribution of chronic pelvic pain to psychological distress.

B

In this prospective cross-sectional study of 149 nulliparous women aged 20–35 with ovarian endometrioma who desired pregnancy, 29.5% had anxiety symptoms, 38.9% had depressive symptoms, and 68.0% of sexually active women had sexual dysfunction symptoms. No statistically significant differences in any psychological outcome were detected between the unilateral and bilateral groups after adjustment for confounders. Chronic pelvic pain, but not cyst laterality, independently predicted both anxiety and depressive symptoms.

C

The study indicates that psychological symptom burden in ovarian endometrioma is substantial regardless of cyst laterality. Chronic pelvic pain, rather than anatomical disease extent, appears to be the more clinically relevant determinant of psychological distress. Routine psychological screening and comprehensive pain management should be offered to all women with ovarian endometrioma.

## Introduction

Ovarian endometrioma (OMA) is a common manifestation of endometriosis, a chronic gynecological disease affecting 10–15% of women of reproductive age and characterized by symptoms such as cyclical dysmenorrhoea, chronic pelvic pain, dyspareunia, irregular bleeding, pelvic adhesions, and infertility [1]. Although the aetiology remains incompletely understood, it is believed to involve autoimmunity, inflammation, genetics, environmental factors, and lifestyle [2].

A substantial body of evidence accumulated over the past decade indicates that women with endometriosis frequently experience symptoms of anxiety and depression [3–8], with prevalence estimates ranging from 9.8% to 98.5% for depressive symptoms and from 11.5% to 87.5% for anxiety symptoms [9]. Sexual dysfunction is also highly prevalent in this population [10]. This psychological burden may, in turn, exacerbate endometriosis symptoms [11] and contribute to a marked decline in quality of life [7].

The question arises as to whether the laterality of OMA (unilateral versus bilateral) is associated with differential levels of psychological distress. Bilateral OMA has been consistently linked to poorer ovarian reserve and reduced fertility potential after surgery compared with unilateral disease [12–21], and a higher risk of disease recurrence [22,23]. Given this greater disease burden, it is plausible that women with bilateral OMA might experience more severe psychological symptoms than those with unilateral disease. However, this association has not been empirically examined, as existing studies comparing unilateral and bilateral endometriosis have focused almost exclusively on ovarian reserve function and fertility outcomes, without assessing psychological endpoints.

Pain, in particular, is one of the most consistent and powerful correlates of anxiety and depressive symptoms in women with endometriosis [24]. Chronic pelvic pain affects a substantial proportion of women with OMA and has been identified as a stronger predictor of psychological distress than anatomical disease severity in several studies. It is therefore essential to account for pain when examining whether disease characteristics such as cyst laterality are independently associated with psychological outcomes.

Despite the plausibility of bilateral OMA conferring greater psychological burden, no prior study has directly compared psychological outcomes between women with unilateral and bilateral OMA. Moreover, the role of chronic pelvic pain as a potential confounder or alternative explanatory variable in this relationship has not been examined.

Therefore, the present study aimed to investigate whether women with unilateral and bilateral OMA differ in anxiety symptoms, after accounting for the presence of chronic pelvic pain. We hypothesized that if cyst laterality independently contributes to psychological distress, women with bilateral OMA would exhibit higher symptom levels than those with unilateral disease, even after adjustment for pain. Conversely, a null finding would indicate that laterality alone is not a meaningful independent correlate of psychological symptoms, and that other factors, particularly pain, warrant greater clinical attention.

## Method and material

### Trial registration

This is a prospective cross-sectional study comparing the anxiety levels between patients with unilateral and bilateral OMA among young nulliparous women. This study has been approved by the ethics committee (II2023-280-01). ChiCTR2300078143, Chinese Clinical Trial Registry (https://www.chictr.org.cn/), date of registration:2023/11/29, date of enrolment of the first subject: 2023/12/01.

### Study population

The research subjects are women of childbearing age who have not given birth but have the desire to conceive, and who are patients with ovarian cysts at the Department of Gynecology of the Third Affiliated Hospital of Sun Yat-sen University, from December 2023 to June 2024.

### Eligibility criteria

The eligibility criteria are shown in Table 1.

**Table 1.** Eligibility criteria.

Inclusion criteria	Exclusion criteria
①Consistent with the clinical diagnosis of endometriosis ovarian cysts;	①patients with a history of anxiety
②Childless and willing to bear children;	②people with neurological and/or cognitive problems, inability to understand the nature and scope of the study, or inability to comply with medical advice;
③20–35 years old;	③patients with chronic pain other than the reproductive system;
④Willing to cooperate with doctors to complete the questionnaire;	④patients with organic lesions.
⑤Signed informed consent.	

### Criteria for suspension and exclusion of cases

(1). Cannot complete the entire questionnaire;
(2). Exhibit severe resistance during the research process;
(3). Patients who are subsequently diagnosed with malignant tumours during follow-up, or patients whose cysts have disappeared and are considered to have physiological cysts;
(4). Withdraw the informed consent form.

### Diagnostic criteria

Since the included cases are first-time patients, a preliminary diagnosis of OMA can be made based on ultrasound diagnosis, which is the most important method for the clinical diagnosis of OMA. For OMA, it can determine the location, size, and shape of the ectopic cysts. Both the diagnostic sensitivity and specificity are above 96% [25].

### Baseline

We recorded patient age, marital status, education, job type, income, sexual debut age, and if it was their first visit as baseline data, to ensure no statistical differences between the groups.

## Observation indicators

### Main observation indicators

The primary outcome measure for anxiety was determined through the application of the Zung Self-Rating Anxiety Scale (SAS) [26].

### Secondary observation indicators

The study’s secondary outcomes include depression status and sexual function scores. Depression is assessed using the Self-Rating Depression Scale (SDS) [27]^.^ The sexual function score comes from the Female Sexual Function Index (FSFI) [28].

### Other indicators

We collected data on patients’ BMI, pelvic mass size, menstrual status, dysmenorrhoea, pelvic pain, treatment delays, fertility plans, infertility status, physician-related factors, and past/current treatment plans to evaluate their effect on anxiety levels.

### Bias control

Since the observation indicators of this study are subjective self-assessment scales, to reduce evaluation bias, this study employed blinding for doctors and data statisticians collecting questionnaires.

### Confirmation diagnosis

A telephone follow-up was conducted three months after enrolment to confirm surgical pathology results or reassess ultrasound diagnoses for endometriosis, ensuring only OMA patients were included in the study.

### Statistical analysis

We collected data using Questionnaire Star, cleaned it in Excel, and analyzed it with SPSS 27.0. We checked for normal distribution and described data accordingly. Count data were presented as frequencies and ratios. We conducted descriptive statistics, compared pre-treatment clinical features, analyzed treatment effects, and assessed outcome factors. All tests were bilateral with *p* < 0.05 indicating significance. We chose statistical methods based on data type and used χ^2^ or Fisher’s exact test for categorization. We applied a t-test for normally distributed data, the Wilcoxon rank-sum test for non-conforming data, and the Mann-Whitney U or Kruskal-Wallis test for rank data comparisons.

After controlling for confounding factors, group (unilateral/bilateral) was tested to determine whether it was independently associated with SAS, SDS, and FSFI scores. Post-hoc power analysis was conducted using G*Power 3.1.9.7. Based on the observed sample sizes and pooled standard deviations, achieved power was calculated for the primary and secondary outcomes at α = 0.05 (two-tailed).

## Result

### Recruitment

The study initially involved 346 individuals, but 293 did not participate for various reasons, including age, health issues, pregnancy, and other factors. After exclusions, 110 patients with ovarian cysts were left, comprising 55 with unilateral and 55 with bilateral cysts. To address non-normal age distribution, the sample size was increased for a second recruitment phase, which was completed regardless of statistical normality.

**Figure 1.**
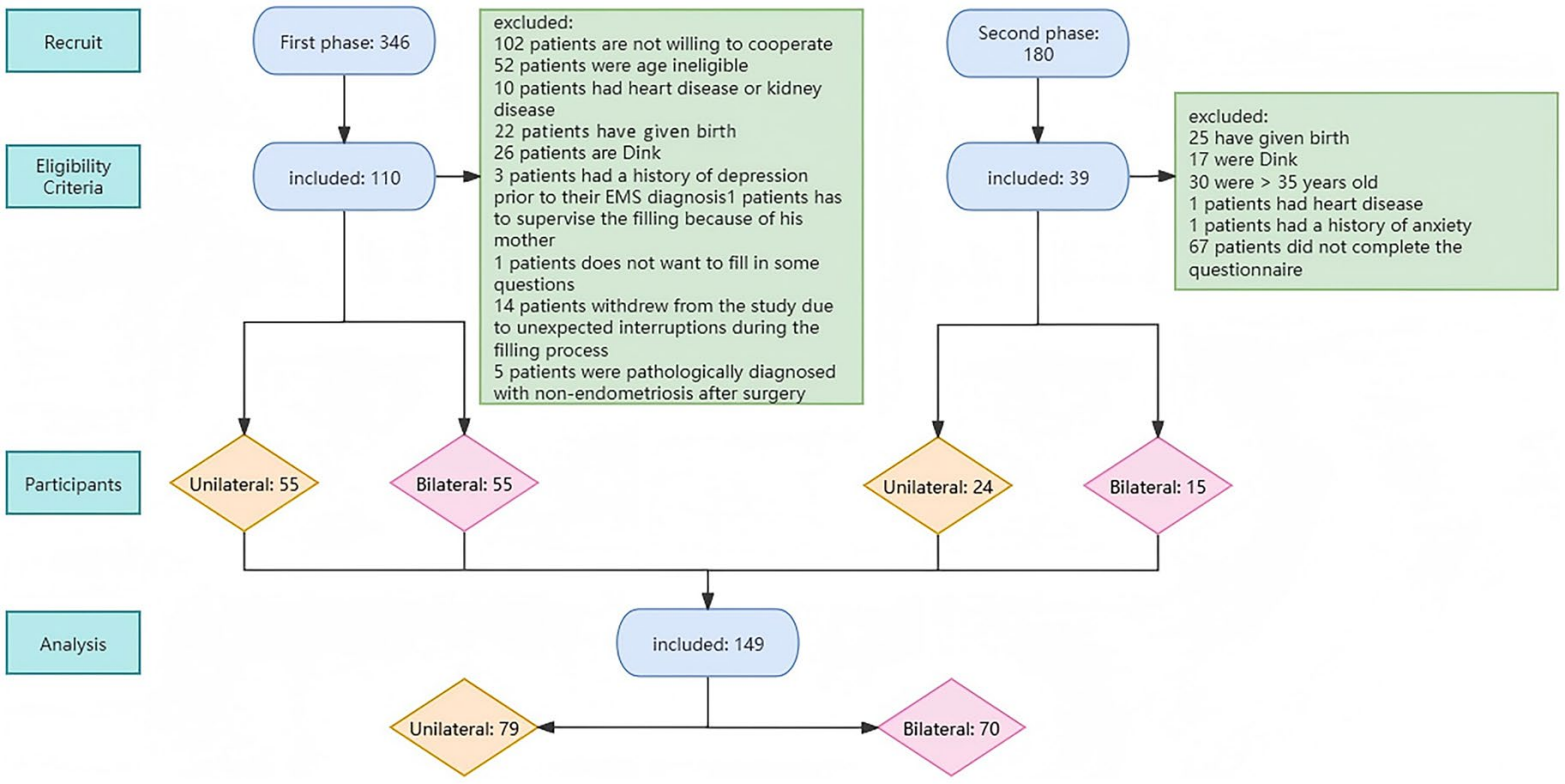
Participant flow chart.

In phase two, 180 participants were initially recruited, but 76 were ineligible for various reasons, leaving 104. Of these, 67 did not complete the interview and questionnaires, resulting in 37 completed sets. After excluding four with other health issues, the study included 33 patients, with 24 having a unilateral cyst and 9 having bilateral cysts. Overall, the study involved 149 patients, including 79 with unilateral cysts and 70 with bilateral cysts (Figure 1).

### Overall situation of all patients

The average age of the patients was 27.45 years with a BMI of 19.46. Most patients (75.2%) held a bachelor’s degree or college education, while 12.8% had a master’s degree or higher. High school or secondary school education was reported by 7.4%, and junior high school education by 4.7%. Mental labour was the primary occupation for 80.5%, with 14.8% combining mental and physical labour, 3.4% being housewives, and 2 individuals engaged in physical labour. Half of the population (53.7%) had sufficient income for basic necessities, while 45.6% were moderately well-off, with 62.4% of these being urban residents. Only 69.1% of patients were sexually active, and 34.2% had non-cardiovascular and cerebrovascular diseases such as chronic pain and breast hyperplasia. Anxiety was present in 29.53% of patients, and 38.92% experienced mild to moderate depression. Among the 103 patients with a history of sexual life, 67.96% reported sexual dysfunction.

### Baseline

There was no significant difference in age, education, job, family income and living environment between patients with unilateral cysts and those with bilateral cysts (Table 2).

**Table 2.** Baseline.

Item	Classification	**Unilateral** (***n* = 79**)	**Bilateral** (***n* = 70**)	*P*
Age		27 (25, 29)	27 25, 29)	0.86^△^
Education	Junior high school	6	1	0.06^△^
	Senior high school	7	4	
	college	58	54	
	Master and doctorate	8	77	
Work	Metal labor	60	60	0.11^△^
	Manual labor	1	1	
	Metal and manual labor	14	8	
	Housewife	4	1	
Family income	<2000	1	0	0.92^△^
	2000–10000	42	38	
	>10000	36	32	
Living environment	rural	50	43	0.75^△^
	urban	8	6	
	other	21	21	
Sexual history	Yes	52	51	0.35^△^
	No	27	19	
First-diagnosis	Yes	22	16	0.48^△^
	No	57	54	

Notes: △: *p* > 0.05 treated as non-significant.

### Disease data

The study revealed a significant difference in cyst count between patients with unilateral and bilateral cysts, with unilateral patients typically having one cyst and bilateral patients having 2–4 cysts. However, no significant differences were found in maximum cyst diameter, endometriosis diagnosis timing, BMI, pregnancy plans, primary infertility, chronic pelvic pain, dysmenorrhoea, dysmenorrhoea duration, or medication use in the past three months (Table 3).

**Table 3.** Disease data.

item	Classification	Unilateral (*n* = 79)	Bilateral (*n* = 70)	*P*
Time of diagnosis (m)		17.06 ± 22.54	22.11 ± 58.18	0.78^△^
BMI		20.56 ± 3.63	19.34 ± 2.21	0.05^△^
Planning for Pregnancy	<6 m	15	8	0.21^△^
	6m-1y	15	11	
	1-3y	10	19	
	3-5y	5	3	
	At a loose end	34	29	
Primary infertility	Yes	8	9	0.60^△^
	No	71	61	
Chronic Pelvic Pain	Yes	40	38	0.66^△^
	No	39	32	
dysmenorrhoea	Yes	52	43	0.58^△^
	No	27	27	
Time of dysmenorrhoea (m)		37.57 ± 46.65	30.26 ± 39.938	0.69^△^
Number of cysts	1	61	1	0.00
	2	15	37	
	3	2	16	
	4	1	10	
	5	0	4	
	6	0	1	
	7	0	1	
Maximum cyst diameter		52.52 ± 31.37	49.70 ± 21.67	0.73^△^
Medication use in the past 3m	Yes	27	34	0.08^△^
	No	52	36	

Notes: △: *p* > 0.05 treated as non-significant; m: month; y: year.

### Anxiety, depression and sexual dysfunction

A t-test was conducted on the SAS, SDS, and FSFI scores of patients with unilateral and bilateral cysts, revealing no significant differences in anxiety, depression, or sexual dysfunction between the two groups (Table 4).

**Table 4.** Outcomes.

Item	Classification	Unilateral (*n* = 79)	Bilateral (*n* = 70)	*P*
SAS	All	46.49 ± 8.02^#^	44.36 ± 7.80^#^	0.10^△^
SASI	<0.5 (no anxiety)	52	53	0.16^△^
	0.5-0.59 (mild anxiety)	22	15	
	0.6-0.69 (moderate anxiety)	5	2	
	>0.7 (severe anxiety)	0	0	
SDS	All	48.50 ± 10.75^#^	46.91 ± 9.50^#^	0.34^△^
SDSI	<0.5 (no depression)	44	47	0.17^△^
	0.5-0.59 (mild depression)	18	13	
	0.6-0.69 (moderate depression)	16	10	
	>0.7 (severe depression)	0	0	
FSFI	All	22.68 ± 5.36^#^	20.91 ± 4.29^#^	0.91^△^
FDS	Yes	36	34	0.87^△^
	No	16	17	

Notes: #: *p* > 0.05 was considered to be in accordance with a normal distribution. △: *p* > 0.05 treated as non-significant.

### Multivariable analysis

After adjusting for age, BMI, chronic pelvic pain, dysmenorrhoea, primary infertility, and medication use in the past three months, no statistically significant associations were found between OMA laterality and any of the three psychological outcomes.

For anxiety symptoms, the bilateral OMA group scored, on average, 2.58 points lower on the SAS than the unilateral group, but this difference was not statistically significant, and the 95% confidence interval narrowly crossed zero (adjusted B = −2.58[−5.19, 0.03], *p* = 0.052). For depressive symptoms, the adjusted group difference was small and non-significant (adjusted B = −1.86[−5.25, 1.53], *p* = 0.279). In the sexually active subsample, FSFI total scores also did not differ significantly between groups after covariate adjustment (adjusted B = −0.91[−2.01, 0.19], *p* = 0.072).

In contrast, chronic pelvic pain emerged as a significant independent predictor of anxiety symptoms (adjusted B=-3.46[−6.04,−0.87], *p* = 0.009) and depression symptoms (adjusted B=-3.86[−7.21,−0.50], *p* = 0.025) (Table 5).

**Table 5.** Multivariable linear regression analyses.

Characteristic	SAS score (*n* = 149)			SDS score (*n* = 149)			FSFI total score (*n* = 103)		
	B[95%CI]	β	P	OR[95%CI]	β	P	OR[95%CI]	β	P
Group(bilateral vs. Unilateral)	−2.58[−5.19, 0.03]	−0.16	0.052	−1.86[−5.25,1.53]	−0.09	0.279	−0.91[−2.01,0.19]	−0.18	0.072
Age(years)	−0.27[−0.65,0.10]	−0.117	0.156	−0.33[−0.82,0.16]	−0.11	0.187	−0.22[−0.56,0.11]	−0.15	0.185
BMI(kg/m^2^)	−0.05[−0.47]	−0.019	0.815	0.08[−0.47,0.62]	0.02	0.783	0.02[−0.37,0.41]	0.10	0.921
Chronic pelvic pain (Yse vs. No)	−3.46[−6.04,−0.87]	−0.22	0.009*	−3.86[−7.21,−0.50]	−0,19	0.025*	−2.10[−4.27,0.07]	−0.21	0.057
dysmenorrhoea (Yse vs. No)	−0.66[−3.32,2.00]	−0.04	0.627	−1.10[−4.57,2.36]	−0.05	0.530	0.06]-2.18,2.29\	0.01	0.961
Primary infertility (Yse vs. No)	−1.68[−5.71,2.36]	−0.07	0.413	−1.48[−6.72,3.76]	−0.052	0.578	0.27[−2.57,3.11]	0.02	0.848
Medication use in past 3 months (Yse vs. No)	−2.06[−4.69,0.57]	−0.13	0.123	−2.04[−5.45,1.38]	−0.046	0.240	−0.29[−2.50,1.93]	−0.03	0.796
Medels R^2^	0.10			0.07			0.09		
Adjusted R^2^	0.05			0.02			0.01		

Note:* *p* < 0.05 represents an independent influencing factor.

Post-hoc power analysis based on the observed effect sizes (Cohen’s *d* = 0.27 for SAS, *d* = 0.16 for SDS, *d* = 0.36 for FSFI) and group sample sizes indicated that the achieved power was 50% for SAS, 25% for SDS, and 51% for FSFI. To detect the observed SAS difference with 80% power, a total sample of approximately 432 participants would be required.

### Sensitivity analysis

To evaluate the representativeness of the sexually active subsample used for FSFI analyses, we compared women who reported a history of sexual activity (*n* = 103) with those who did not (*n* = 46). The two groups did not differ significantly in age, education, anxiety symptoms (SAS), depressive symptoms (SDS), chronic pelvic pain, dysmenorrhoea, or laterality of OMA (all *p* > 0.05). The only statistically significant difference was in maximum cyst diameter, which was larger in the non-sexually active group (*p* = 0.023). These findings suggest that the sexually active subsample was broadly comparable to the overall cohort, supporting the validity of the FSFI analyses within this subgroup. Detailed results are presented in Supplementary Table S1. (Differential analysis between patients with sexual history and non-sexual history)

## Comment

### Principal findings

This prospective cross-sectional study investigated anxiety symptoms, depressive symptoms, and sexual dysfunction symptoms among 149 nulliparous women aged 20–35 with OMA who expressed a desire for pregnancy. Three principal findings were delineated. First, the symptom burden was considerable: approximately one-third of the women reported mild-to-moderate anxiety symptoms, nearly two-fifths reported mild-to-moderate depressive symptoms, and over two-thirds of sexually active participants reported symptoms of sexual dysfunction. Second, no statistically significant differences were observed in any psychological outcomes between women with unilateral and bilateral OMA, either in univariable analyses and after multivariable adjustment. Third, and most notably, chronic pelvic pain, but not cyst laterality, was identified as a significant independent predictor of both anxiety and depressive symptoms in the adjusted models.

### Results in the context of what is known

The elevated prevalence of anxiety, depressive, and sexual dysfunction symptoms in our sample is consistent with prior literature on endometriosis [3,9]. Approximately one third of patients showed mild-to-moderate anxiety symptoms, and nearly one half showed mild-to-moderate depressive symptoms. About two thirds of sexually active women have sexual dysfunction. It should be noted that these data were collected from women who voluntarily completed sensitive psychological and sexual function questionnaires, meaning the actual population prevalence may be even higher.

The central novel finding of this study is the absence of a detectable independent association between OMA laterality and psychological symptom severity. Previous comparisons of unilateral and bilateral endometriomas have focused almost exclusively on ovarian reserve and surgical outcomes. Studies by Wang et al. found that serum AMH concentrations after cystectomy were higher for unilateral than for bilateral cysts [29], and that older age and bilateral cysts were risk factors for AMH decline after surgery [12]. Karadağ et al. and Kovačević et al. similarly reported lower AMH levels in bilateral OMA patients compared with unilateral OMA patients [13,14]. Nguyen et al. demonstrated that AMH levels remained lower 12 months postoperatively in bilateral cyst patients [15], and Ghazal et al. identified bilateral endometriosis as a risk factor affecting postoperative AMH [16]. Nankali’s 2020 meta-analysis showed lower AMH levels after bilateral versus unilateral cystectomy, though without statistical analysis [17]. Youins’ 2019 meta-analysis indicated bilateral laparoscopic surgery has lasting harmful effects on ovarian reserve [18].

Regarding recurrence, Chung et al. showed that the recurrence rate after laparoscopic cystectomy was significantly higher for bilateral cysts [19], and Roman et al. confirmed that bilateral ovarian cysts are the only risk factor for postoperative recurrence in endometriosis [20]. Additional evidence suggests that bilateral endometriomas are associated with greater surgical difficulty and more extensive extra‑ovarian disease [21,22].

Given this well‑documented impact of bilateral OMA on ovarian reserve and recurrence risk, we had anticipated that women with bilateral cysts might experience greater psychological distress, mediated by heightened concerns about future fertility. Our data do not support this hypothesis. However, it is important to note that two studies that did not find significant differences in AMH or IVF outcomes between unilateral and bilateral endometrioma patients included participants who had not undergone surgery [23,30], raising the possibility that surgical intervention itself may be a critical modifier of the relationship between laterality and ovarian function.

### The central role of chronic pelvic pain

The null association between laterality and psychological outcomes necessitates interpretation within the context of pain’s pivotal role. In our multivariable analyses, chronic pelvic pain emerged as an independent correlate of both anxiety and depressive symptoms, whereas cyst laterality did not demonstrate a significant relationship. This finding implies that the subjective experience of pain, rather than the anatomical burden of ovarian disease, may constitute a more proximate determinant of psychological distress in individuals with ovarian endometrioma. This perspective aligns with accumulating evidence underscoring pain as one of the strongest correlates of anxiety and depression in endometriosis. It is also consistent with the illness representations framework [31], which proposes that objective disease characteristics do not directly translate into psychological symptomatology but are mediated through patients’ subjective interpretations, among which pain is predominant.

The attenuation of any potential association between laterality and anxiety following adjustment for pain also carries conceptual implications. This suggests that if bilateral OMA indeed elevates psychological distress, the effect might be mediated indirectly *via* heightened pain severity or more intricate pain phenotypes, as opposed to a direct cognitive pathway entailing anticipated infertility. However, the pain assessment was limited to the presence or absence of chronic pelvic pain and dysmenorrhea, and we did not measure pain severity, pain interference, pain duration, or pain-related cognitive processes such as catastrophizing. These unmeasured pain dimensions may be important in fully characterizing the pain-psychology relationship.

### Interpretive frameworks and future directions

Several broader interpretive frameworks may help contextualize our findings, although none were directly tested in this study and should be regarded as hypotheses for future investigation.

First, the illness representations framework proposes that objective disease characteristics do not automatically translate into psychological symptoms; rather, their impact is shaped by individuals’beliefs about their condition and other psychosocial processes [32,33]. In the context of our findings, this framework suggests that women with bilateral OMA may not necessarily perceive their condition as more threatening than those with unilateral disease, particularly if they have not received fertility counselling or have not yet attempted conception. Because we did not measure illness perceptions, fertility-related distress, or perceived infertility threat, we cannot evaluate whether these cognitive pathways mediate or moderate the laterality–anxiety relationship.

Second, emerging evidence points to shared biological substrates between endometriosis and psychological symptoms. Tian et al. found that differential gene expression in multiple brain regions of mice with endometriosis induced pain sensitization, anxiety-like behaviour, and depressive-like behaviour [34], while Bashir et al. demonstrated central nervous system-wide glial activation in a mouse model of endometriosis [35]. The observation that chronic pelvic pain independently predicted both anxiety and depressive symptoms in our study is compatible with the hypothesis that these conditions share common mechanisms, including HPA axis dysregulation, augmented inflammation, altered immunity, and central sensitization [6,34–40]. If such biological pathways operate relatively independently of anatomical disease extent, they could produce uniformly elevated psychological distress across laterality subgroups, consistent with our null findings. However, we did not measure any biological markers, so this interpretation remains speculative.

Third, fertility-related concerns and clinical communication practices may play a role that our study could not capture. Jourdain et al. reported that 74% of physicians would offer fertility advice to patients with bilateral cysts, compared with only 33% for unilateral cysts [41], suggesting that differential counselling may shape patient perceptions. Whether such differences in clinical communication translate into differential psychological outcomes remains an open question.

Several priorities for future research emerge from these considerations. Larger prospective studies with adequate statistical power are needed to detect small-to-moderate effects that the present sample was underpowered to identify. Our post-hoc power analysis indicated that to detect the observed difference in SAS scores with 80% power would require approximately 432 participants. Subsequent research should directly assess fertility-related distress, illness perceptions, perceived social pressure, and pain-related cognitive processes (e.g. catastrophizing, pain interference), in order to formally test mediational pathways that could not be examined here. The inclusion of biological markers, such as inflammatory cytokines, hormonal profiles, or neuroimaging indices, would enable the simultaneous examination of physiological and psychosocial mechanisms within a unified biopsychosocial framework. Such integrative designs are essential to move beyond the unproductive dichotomy of “physiological versus social” causes and towards a more nuanced understanding of psychological distress in endometriosis.

### Clinical implications

These findings yield several clinical implications. At the most immediate level, they indicate that psychological screening ought to be routinely administered to all women with OMA, regardless of cyst laterality. The uniformly elevated prevalence of anxiety, depressive, and sexual dysfunction symptoms demonstrates that neither unilateral nor bilateral disease should be regarded as “low risk” for psychological morbidity.

Beyond screening, the robust and independent association between chronic pelvic pain and psychological symptoms underscores the centrality of comprehensive pain management in alleviating psychological distress. Sole focus on cyst characteristics, without addressing the subjective pain experience, may prove inadequate for identifying or effectively treating those at highest psychological risk. Multidisciplinary strategies integrating gynaecologic pain management, psychological support, and sexual health services are likely to confer the greatest benefit.

More broadly, the observation that bilateral OMA was not independently associated with heightened anxiety implies that clinicians should not presume women with more extensive anatomical disease are necessarily more distressed, nor that those with unilateral disease are psychologically well. Individualized assessment remains imperative.

### Strengths and limitations

This study has several strengths. It is among the first to directly compare psychological outcomes between women with unilateral and bilateral OMA. The prospective design ensured standardized data collection using validated instruments. The sample size, while modest, was reasonably balanced between groups. The inclusion of multivariable analyses adjusted for key confounders (particularly chronic pelvic pain) strengthens the internal validity of our findings.

Several limitations warrant consideration. First, the cross-sectional design does not allow for assessment of psychological changes and causal inferences. Second, excluding patients with prior anxiety or depression may have reduced outcome variability and biased comparisons, limiting conclusions to women without psychiatric history. Third, high non-participation and incomplete questionnaires may cause selection bias; sensitivity analysis showed only cyst diameter differed between sexually active and inactive groups, but selection effects cannot be excluded. Fourth, restricting to nulliparous women aged 20–35 desiring pregnancy limits generalizability, especially for women near 40. Fifth, OMA diagnosis relied mainly on ultrasound with partial surgical confirmation, possibly leading to exposure misclassification, which the 3-month follow-up may reduce. Sixth, post-hoc power analysis indicated limited power to detect small-to-moderate effects; the non-significant difference does not imply equivalence, and a clinically meaningful difference cannot be excluded. Seventh, pain assessment was limited to binary clinical variables and did not capture pain severity, interference, or pain-related cognitive processes. Finally, the study did not measure key psychosocial constructs (fertility-related distress, illness perceptions, perceived stigma) or biological markers; therefore, the relative contributions of physiological, psychological, and social factors to OMA-related psychological distress could not be evaluated.

## Conclusion

In this investigation, after adjusting for key confounders including chronic pelvic pain, no statistically significant differences in symptoms of anxiety, depression, or sexual dysfunction were observed between individuals with unilateral and bilateral ovarian cysts. Chronic pelvic pain was identified as a significant independent predictor of both anxiety and depressive symptoms. These findings indicate that pain, as opposed to the laterality of anatomical disease, may constitute a more clinically relevant determinant of psychological distress in OMA, underscoring the necessity of comprehensive pain assessment and multidisciplinary care within this population.

## Supplementary Material

supplementary table S1.doc

## Data Availability

The datasets generated during the current study are available. It will be shared in ClinicalTrials.gov. The authors confirm that the data supporting the findings of this study are available within the article and its supplementary materials.

## Attestation statement

- Data regarding any of the subjects in the study has not been previously published unless specified.
- Data will be made available to the editors of the journal for review or query upon request.
