# Supplementary material for: No difference in anxiety symptoms between women with unilateral and bilateral ovarian endometriosis: a prospective cross-sectional study
Source: Ann Med. 2026 Aug 4;58(1):2711545. doi: 10.1080/07853890.2026.2711545 (PMC13439771; doi:10.1080/07853890.2026.2711545)
Supplement: Supplementary file 1 [file IANN_A_2711545_SM1953.doc]

Supplementary table S1 Differential analysis between patients with sexual history and non sexual history

| **Item** | **classification** | **Sexual history**  **（n=103）** | **Non sexual history**  **（n=46）** | ***P*** |
| --- | --- | --- | --- | --- |
| Age |  | 27(25,29) | 27(25,31) | 0.984 |
| Education | Junior high school | 7(6.8%) | 0(0.0%) | 0.309 |
|  | Senior high school | 8(7.8%) | 3(6.5%) |  |
|  | college | 76(73.8%) | 36(78.3%) |  |
|  | Master and doctorate | 12(11.7%) | 7(15.2%) |  |
| Work | Metal labour | 84(81.6%) | 36(78.3%) | 0.301 |
|  | Manual labour | 1(1.0%) | 1(2.2%) |  |
|  | Metal and manual labour | 13(12.6%) | 9(19.6%) |  |
|  | Housewife | 5(4.9%) | 0(0.0%) |  |
| Family income | ＜2000 | 1(1.0%) | 0(0.0%) | 0.427 |
|  | 2000-10000 | 52(50.5%) | 28(60.9%) |  |
|  | ＞10000 | 50(48.5%) | 18(39.1%) |  |
| Living environment | rural | 66(64.1%) | 27(58.7%) | 0.415 |
|  | urban | 11(10.7%) | 3(6.5%) |  |
|  | other | 26(25.2%) | 16(34.8%) |  |
| First-diagnosis | Yes | 22(21.4%) | 16(34.8%) | 0.082 |
|  | No | 81(78.6%) | 30(65.2%) |  |
| Time of diagnosis（m） | \ | 10(3,24) | 6.5(3,15.25) | 0.223 |
| BMI | \ | 19.13(17.69,20.69) | 19.78(18.06,22.79) | 0.123 |
| Chronic Pelvic Pain | Yes | 51(49.5%) | 27(58.7%) | 0.300 |
|  | No | 52(50.3%) | 19(41.3%) |  |
| dysmenorrhea | Yes | 66(64.1%) | 29(63.0%) | 0.903 |
|  | No | 37(35.9%) | 17(37.0%) |  |
| Time of dysmenorrhea（m） | \ | 13(6,48) | 12(6,48) | 0.928 |
| Number of cyst | \ | 2(1,3) | 1.5(1,2) | 0.061 |
| Maximum cyst diameter | \ | 48(28,63) | 57.50(44.75,73.25) | 0.023^△^ |
| Medication use in the past 3m | Yes | 42(40.8%) | 19(41.3%) | 0.952 |
|  | No | 61(59.2%) | 27(58.7%) |  |
| SAS score | \ | 45.00(41.25,50.00) | 44.38(38.75,50.31) | 0.419 |
| SDS score | \ | 47.50(42.50,56.25) | 43.75(38.44,51.88) | 0.119 |

Notes：△：*P<*0.05: the difference between the two groups was statistically significant. m: month.
